# The Formula of Humanity and AI Use

**DOI:** 10.1007/s13347-026-01100-w

**Published:** 2026-05-01

**Authors:** Martin Sticker

**Affiliations:** University of Bristol, Bristol, United Kingdom

**Keywords:** AI, Kant, Exploitation, Formula of humanity, Work

## Abstract

Aylsworth and Castro have argued that, following Kant’s Formula of Humanity, using ChatGPT to write humanities essays constitutes a violation of a duty to cultivate one’s humanity. I first turn to a critical evaluation of their argument and then point to a further dimension in which the FH has a bearing on the ethics of AI use. My positive contribution is to propose that Kant’s Formula of Humanity can contribute to the ethics of LLM use when we focus on the prohibition against treating *others* as mere means. Kant’s practical philosophy is in an excellent position to capture both the danger of treating others as mere means and to account for the value of being a means. AI may endanger both: the status of some workers as ends rather than mere means and the ability of workers to function as ends on their own terms.

## I

Aylsworth and Castro (2024) argue that ChatGPT use can deprive students of significant opportunities to cultivate their humanity and can thus violate a duty to self. To make their case they draw on Kant’s Formula of Humanity (FH), which mandates that “*you use humanity*,* whether in your own person or in the person of any other*,* always at the same time as an end*,* never merely as a means*” (IV:Kant, 1900) 429.10-2).

Their argument is that:

1. You have a duty to cultivate your humanity, because this capacity has final value.
2. If you have a duty to cultivate something, happen upon a good and unique opportunity to cultivate it, and do not have a good reason to pass on the opportunity, you ought to take the opportunity.
3. Writing your own humanities papers is one such opportunity.
4. So, you ought to write your own humanities papers. (Aylsworth & Castro, 2024, p. 20)

There is much to be commended about this argument. Firstly, Aylsworth and Castro’s approach neither condemns nor licenses AI use wholesale. Rather, the ethics of AI use depends on what the task is that is delegated to an AI. Secondly, Aylsworth and Castro bring the FH to bear on AI ethics, and I hope that their paper will be the start of an in-depth discussion of AI ethics from a Kantian perspective.

In my current note, I want to follow Aylsworth and Castro’s lead and first turn to a critical evaluation of their argument (2) and then point to a further dimension in which the FH has a bearing on the ethics of AI use (3).

## II

There are two problems with Aylsworth and Castro’s argument.

### Humanity is Ambiguous

Aylsworth and Castro characterize humanity as “your capacity to self-govern” (p.13), or to set and pursue your own ends. I propose that we refer to this sense of humanity as *self-governance*. Whilst self-governance is Aylsworth and Castro’s official account of humanity there is a second sense of humanity at play in their paper, namely, as *reasoning*. When explaining what is special about essays in the humanities, Aylsworth and Castro point out that these are essays “in which you are charged with giving or evaluating reasons for or against a non-trivial conclusion (e.g., that we have free will, that it is morally wrong to eat meat, etc.)” (p.2). Moreover, as examples for “capacities that are essential to autonomy”^1^ they suggest “our ability to weigh reasons, think through problems” (p.20). However, I can reason about non-trivial conclusions, weigh reasons and think through problems that have nothing to do with *my* end-setting or self-governance.

In premise 1 Aylsworth and Castro claim that humanity has final value in the sense that it is “the terminal value in the chain of justification” and that “the terminal value really is authoritative” (p.11). However, Aylsworth and Castro’s argument for the final value of humanity tacitly assumes humanity as *reasoning*:

Suppose you are asked why we should not value humanity. What would you do? Presumably, you would give reasons to think that humanity *isn’t* valuable. By doing this, you betray yourself: you’ve just *used* your capacity to evaluate ends and have thereby *endorsed it* in using it in your defense (p.14).

Aylsworth and Castro claim that what you would be doing here is to use your capacity to evaluate an end, namely, humanity, thus presenting this as an exercise of humanity as self-governance. However, in their scenario you are not reflecting about what ends *you* ought to pursue, but rather about a theoretical or metaphysical question. From the capacity for reasoning being inevitable for settling theoretical or metaphysical questions, and thus being of final value, it does not follow that self-legislation does have the same value.^2^

One response to this ambiguity would be to drop the attempt to present an irrefutable argument that humanity has final value. Aylsworth and Castro themselves stress that such an argument is essential, since other arguments “fail to ground their claims in an educational objective that has final value” (fn.4). However, they in fact put an unnecessarily heavy burden of proof on themselves here. For their argument to be successful Aylsworth and Castro merely need to maintain that humanity is valuable, and we therefore have a duty to cultivate it. They could thus drop appeal to humanity as reasoning and focus on self-governance. Moreover, there already exist a number of other arguments to the effect that writing is a form of thinking or reasoning and our capacity for reasoning may therefore be undermined when writing is delegated to an AI (Mintz, 2021; Gerlich, 2025). I therefore think that it would benefit Aylsworth and Castro’s overall argument to give up the attempt to vindicate premise 1 via an appeal to the inevitability of reasoning and focus their argument on self-governance.

Finally, Aylsworth and Castro’s premise 3 proposes that humanities essays are a good and unique opportunity to cultivate humanity. However, if premise 1 were, in fact, concerned with humanity as reasoning then it is not clear how unique such opportunities really are, since there are presumably many other opportunities for developing one’s reasoning capacity. This leads directly to the second problem, the question of how to understand the notion of a salient opportunity to promote one’s humanity.

### A Duty to Study the Humanities?

The duty to cultivate our humanity is an imperfect duty, which requires that agents adopt and further certain ends. There is debate in the Kant literature about whether imperfect duties allow for latitude to do less than the maximum as long as agents do enough and act in all particularly salient cases (see e.g. Hill, 1992). Aylsworth and Castro think there is such latitude (Aylsworth & Castro, 2024, p. 17–18). You only have to take the opportunity to cultivate your humanity if you come across a “a good and unique opportunity” to do so, and do not have a “good reason to pass on the opportunity” as they state in premise 2.

Due to their latitudinarian approach Aylsworth and Castro need to maintain that humanities essays create a special context for cultivation of one’s humanity, such that an agent cannot simply pass on the opportunity such essays afford. They therefore emphasis that humanities essays afford opportunities “that are precious, and you are not likely to encounter them again” (p.19). Students receive feedback on these essays from professionals, and students write them at a critical juncture of their lives, namely, when they are deliberating about what ends (careers and other pursuits) to dedicate their lives to (p.18–20).

However, if it is a violation of duty to self to pass on *one* opportunity to write a humanities essay, because these essays create a particularly salient context for the exercise of self-perfection, then this would be even more so the case if someone passes up on the opportunity to study a humanities *degree*. After all, such a degree would provide many unique or precious opportunities to cultivate humanity. Yet, a duty to study a humanities degree is hardly plausible. I will not further justify this assumption here other than by pointing out how odd it would be to think that someone who chooses to study medicine would thereby be violating a duty to self.

Aylsworth and Castro could reply that other degree choices (and other career choices) equally provide opportunities to cultivate humanity. They indeed concede that their argument “could be extended to students in other disciplines, at community colleges and even pre-college settings, such as high school” (p.21). However, if we understand humanity as self-governance, as we should, it is not clear that assessments in other disciplines do constitute good and unique opportunities to develop this capacity.

Moreover, if many other activities did constitute good and unique opportunities to develop one’s humanity (for instance solving mathematical equations, learning how to do empirical research, working with data or with tools, developing social skills) then it may turn out that many cases of delegating humanities essays to an AI are innocuous if the student instead cultivated their humanity in one of the many other ways open to them (socialized with friends and thereby developed social skills, pursued an extracurricular interest in mathematics, etc.). I take it therefore that Aylsworth and Castro would need to maintain that there is something special about humanities essays and that the opportunities other degrees and pursuits provide are not equally good ways to develop one’s humanity. However, in that case their argument may imply an implausible duty to study the humanities.

## III

I think there is an alternative route for Kantians to speak to the ethics of AI use. This route focuses on how *others’* humanity may be undermined by AI use.

There are at least two versions of this problem.^3^ Firstly: "workers in regions such as Nigeria and Kenya have been required to engage with offensive, traumatic, or otherwise harmful content to refine AI models, while earning wages as low as $1.50 per hour and lacking adequate labor protections." (de Fine Licht, 2025, p. 3). Kantian ethics, and the FH in particular, is already an established source for critical debates about exploitation of workers. Sweatshop-like working conditions are paradigm cases for treatment as mere mans and failure to treat workers as ends in themselves (e.g. Arnold, & Bowie, 2003). Kantian discussions about unjust treatment of workers and others are frequently supplement by a notion of complicity and potential duties of reparation (see e.g. Pogge, 2002, Williams, 2019). After all, most people in developed countries do not own or manage sweatshops. Yet, they face the question of whether it is ethical to use goods produced and services rendered by workers in sweatshops. A very similar question may pertain to AI use if workers who contributed to developing the AI were treated as mere means in the process.^4^ Instead of going into more detail about this, I want to look at a second problem, one that is more distinctive of AI.

Secondly, Anthropic recently settled a class action lawsuit for $1.5 billion, in which it was alleged that:

"Anthropic has built a multibillion-dollar business by stealing hundreds of thousands of copyrighted books". (Bartz et al. 2024, § 1)

"Anthropic’s Claude LLMs compromise authors’ ability to make a living, in that the LLMs allow anyone to generate—automatically and freely (or very cheaply)—texts that writers would otherwise be paid to create and sell." (ibid.§ 4)

There are two separate allegations here. The first one is copyright infringement.^5^ The second is that Claude undermines author’s ability to make a living. The two allegations are related. Claude can generate texts that undermine author’s abilities to make a living not because Claude competes with authors on a level playing field and offers superior products. Rather, “Anthropic’s immense success has been built, in large part, on its largescale copyright theft” (ibid.§ 20).

Kant’s practical philosophy is particularly well placed to account for what may strike us as galling about the wrongdoing that is alleged here. A number of Kantians have recently emphasized that Kant does acknowledge that being a means has positive ethical significance, if it happens on one’s own terms and not as a mere means (Mieth & Williams, 2023; Sticker, 2025).^6^ Being a means for others can take the form of working a useful job, which is both good for others and society at large,^7^ and allows workers to gain material independence and self-esteem via their work.^8^ Being deprived of opportunities to be a means can be understood as a form of exclusion or marginalization, since being able to function as a means on one’s own terms for ends of one’s choosing is a central form of participation in society. Marginalization of this kind is, for instance, experienced by asylum seekers in the UK who lack the right to work or, in other words, to function as a paid means for employers (Mieth & Williams, 2023). Such treatment is incompatible with being treated as an end in oneself. A central aspect of what it means to be treated as an end, or to be self-governing, is the freedom to decide which purposes you want to pursue or what purposes, people or organizations you want to function as a means (albeit never a mere means) for.

Kant’s practical philosophy is in an excellent position to capture both the danger of treating as mere means and to account for the value of being a means. AI may endanger both: the status of some workers as ends rather than mere means and the ability of workers to function as means on their own terms. Discussions about how technology may displace workers or undermine valuable elements of work are currently ubiquitous.^9^ The cases Kant’s practical philosophy can account for particularly well are those where workers may be displaced due to preceding injustices inflicted on them (copy-right infringement) or on other workers (exploitation). Moreover, Kant is not worried about meaningful work per se, but rather about workers’ capacity to set their own ends, including the end to make a living in a field of their choosing.

Focusing on how AI impacts *others* humanity raises at least two points that require further discussion.

Firstly, Kantians need to present a model of responsibility and complicity specifically for AI use. If I use an AI to write an essay or for any other purpose I am not treating anyone as a mere means directly. Rather, I may benefit from exploitative labour that went into the training of the AI as well as from potential copyright infringements. I would also participate in replacement of workers if I delegate to an AI a task that otherwise I would pay a professional for. This may give me moral reasons or even a duty to not use certain AI models or not use AIs for certain tasks.

Secondly, Kantians have to think more about what it means to function as a means for others especially in the context of paid work. After all, a person is not free to take up any job they like. For instance, they may lack the qualifications to practice as a doctor. Moreover, in the past many occupations have disappeared because of technological innovation, and this was not necessarily a bad thing. A nuanced Kantian discussion of the ethics of AI use requires a better understanding of the relation between self-governance or end-setting, being a means and work. This touches upon the, already much discussed, questions of the potential intrinsic value of certain jobs and of work in general. It also requires us to think about whether it may be particularly morally problematic if certain opportunities to work are being foreclosed due to unfair and unjust practices, such as exploitation or copyright theft.^10^

## IV

The FH is a compelling principle to understand the ethics of our engagement with AI. One way of spelling out the FH’s fruitfulness are imperfect duties to self. I have explained that Aylsworth and Castro’s argument contains an important ambiguity. I have also argued that their argument may entail a duty to study a humanities degree, which I consider implausible. Whilst I do not mean to deny that AI use raises significant questions about personal development and our capacity for critical thinking, I think a more pertinent FH-based concern is how AI use reflects on our treatment of *others*. In using AI, we may benefit from treatment of certain workers as mere means. We may also benefit from and participate in a process that deprives others of the opportunity to be a means on their own terms. This may be particularly problematic if such opportunities are not lost as the result of fair competition.

My focus on duties to others pertains to many forms of AI use, not merely to use for humanities essays. I think that this is a virtue of my proposal. Obviously, there may be additional reasons, such as deception and duties to self, for why AI should not be used specifically for writing student essays.

## Data Availability

Not applicable.

## Footnotes

1 Aylsworth and Castro use “humanity” and “autonomy” interchangeably (p.13 − 4), and in the sense of personal autonomy, i.e., not in Kant’s own moralized sense (p.13, fn.23).

2 Kant himself is aware of the distinction at least in the sense that he entertains the possibility that the “most rational being of the word”, an entity capable of perfect reasoning, may lack the moral incentive or self-legislation in the Kantian sense (VI:23fn). Such an entity is able to reason about metaphysical and theoretical matters but not able to self-legislate.

3 I here bracket a further version: AI use as a form of “deception in ways that disrespect the humanity of your professor” (Aylsworth and Castro p.20). Whilst I do think that the deceptive element is significant for academic contexts (e.g. as a rationale for why students should be penalized for AI cheating) the two problems I will be focusing on strike me as more significant from a societal and justice perspective.

4 This is not to deny that many workers involved in developing AI, such as highly skilled software engineers, are not exploited.

5 The problem is by no means limited to Anthropic. See for instance a lawsuit by *The New York Times* against OpenAI (The New York Times 2023).

6 This is most apparent when Kant characterizes the *Kingdom of Ends* as agents relating “to one another, as ends and means” (IV:433.26-33). Being an end *and means* is how people exist for each other in Kant’s ethical ideal.

7 In fact, Kant thinks that “a human being has a duty to himself to be a useful member of the world” (VI:446.1-3).

8 Kant particularly stresses the significance of being able to “work one’s way up” (VI:315.13-22), though this is certainly not the only benefit of work (Gheaus & Herzog, 2016).

9 See *pars pro toto* Danaher (2017).

10 There are also significant questions about the nature of copy-right. For instance, if AI companies reached agreements with publishing companies who legally own copyrights, could it still be the case that authors are treated unfairly, if they were not consulted adequately? This may for instance be the case for academics who have to relinquish their copyrights when publishing with certain publishers.
